# Probiotic–Herb–Peptide Combined Formula Alleviates Alcoholic Liver Damage Associated with Modulation of the Gut–Liver Axis and Ferroptosis

**DOI:** 10.3390/nu18172790

**Published:** 2026-08-26

**Authors:** Yining Wang, Jingyang Tong, Weilong Liu, Chao Huo, Xuegang Luo

**Affiliations:** Key Laboratory of Industrial Fermentation Microbiology of the Ministry of Education and Tianjin Key Laboratory of Industrial Microbiology, College of Biotechnology, Tianjin University of Science and Technology, Tianjin 300457, China; wyning620@163.com (Y.W.);

**Keywords:** functional nutritional formulation, acute alcohol intoxication, gut microbiota, ferroptosis, short-chain fatty acids, AMPK signaling

## Abstract

**Background:** Acute alcohol intoxication causes liver injury and delays recovery via oxidative stress, ferroptosis, inflammation, and gut dysbiosis. This study investigated whether pretreatment with an Alcohol Relief Formula (ARF), composed of soybean peptides, botanical extracts, and probiotics, protects against alcohol-induced liver damage in a preventive model and explored the underlying mechanisms. **Methods:** Mice with acute alcohol intoxication were pretreated with ARF. Liver damage, hepatic alcohol-metabolizing enzymes, antioxidant capacity, inflammatory cytokines, and lipid peroxidation were measured. Ferroptosis markers and AMP-activated protein kinase (AMPK) signaling were assessed. Gut microbiota composition and cecal short-chain fatty acids (SCFAs) were analyzed. Correlations between SCFAs, ferroptosis suppression, and AMPK activation were evaluated. **Results:** ARF pretreatment alleviated acute liver damage and accelerated sobriety. It enhanced alcohol dehydrogenase (ADH) and aldehyde dehydrogenase (ALDH) activities, inhibited CYP2E1 expression, and improved alcohol metabolism. ARF pretreatment restored antioxidant capacity, decreased pro-inflammatory cytokines and lipid peroxidation, and ameliorated hepatic histopathological changes. Notably, ARF pretreatment was associated with suppressed ethanol-induced ferroptosis markers, including increased glutathione peroxidase 4 (GPX4), solute carrier family 7 member 11 (SLC7A11), and ferroptosis suppressor protein 1 (FSP1) expression, elevated AMPK phosphorylation, and reduced hepatic total iron content. In parallel, ARF pretreatment was associated with modulation of the alcohol-disrupted gut microbiota, increased short-chain fatty acid (SCFA)-producing bacteria, and maintained cecal acetate, propionate, and butyrate levels. Correlation analysis revealed that SCFA restoration was positively associated with ferroptosis marker modulation and AMPK activation, suggesting a potential link between gut metabolites and hepatic protection. **Conclusions:** ARF may serve as a potential preventive nutritional strategy for acute alcohol intoxication by promoting recovery and mitigating liver damage in this acute binge pretreatment model. The observed effects may involve the modulation of gut microbiota-derived SCFAs and AMPK signaling associated with ferroptosis markers.

## 1. Introduction

Unrestricted alcohol use can result in alcoholic liver disease (ALD), progressing to alcoholic hepatitis and alcoholic fatty liver, as well as to liver cancer and alcoholic cirrhosis in extreme situations [1]. In recent years, unrestricted alcohol intake has been a major health concern globally. Every year, alcohol-related causes claim the lives of over 3.3 million individuals around the world, and this number is rising [2]. In China, the pooled prevalence of ALD is estimated at 4.8% (95% CI, 3.6–6.2%), with a notably higher rate in men (9.3%) than in women (2.0%) [3]. The proportion of regular drinkers among Chinese adults has risen dramatically from 27.0% in 2000 to 66.2% in 2015, accompanied by an increase in heavy drinking [3]. These trends have made acute alcohol intoxication (AAI) and its associated liver injury an increasingly pressing public health issue. AAI refers to an abrupt and notable rise in ethanol levels in the blood caused by excessive intake of alcohol over a brief duration [4]. The stomach, liver, and kidneys are severely affected by this condition, which in extreme circumstances could be fatal [5]. The gastrointestinal tract absorbs alcohol after intake, and the systemic circulation distributes it throughout the body. It is principally metabolized by the liver, generating 80% to 90% of the enzymes that control alcohol metabolism [6].

In the liver, aldehyde dehydrogenase (ALDH), alcohol dehydrogenase (ADH), and cytochrome P450 family 2 subfamily e polypeptide 1 (CYP2E1) are the main enzymes that drive ethanol metabolism; catalase is a secondary pathway [7]. Most ethanol is oxidized by ADH to acetaldehyde, and ALDH oxidizes it to acetate [8]. Following conversion into acetyl-CoA, acetate enters the tricarboxylic acid (TCA) cycle and is ultimately oxidized to carbon dioxide and water. Beyond this canonical metabolic route, ethanol exposure impairs the hepatic antioxidant defense system by reducing glutathione (GSH) biosynthesis, which diminishes cellular antioxidant capacity and drives excessive reactive oxygen species (ROS) accumulation [9]. Concurrently, alcohol disrupts hepatic iron homeostasis by downregulating hepcidin, a key regulatory hormone of iron metabolism, which impairs iron transport and utilization and results in elevated intracellular iron levels [10,11]. The combined effect of oxidative stress and intracellular iron overload serves as the primary trigger for ferroptosis. In addition to direct hepatocellular damage, prolonged or heavy alcohol intake also exerts detrimental effects on the gastrointestinal tract, where the gut microbiota—often referred to as the human “second genome”—plays a pivotal role in gut–liver axis crosstalk [12,13]. Alcohol-triggered dysbiosis and elevated permeability of the gut disrupt gut–liver interaction [14]. Intestinal and hepatic damage are worsened by hazardous acetaldehyde from alcohol metabolism and infectious microbial compounds that seep into the circulation [15].

AMP-activated protein kinase (AMPK) regulates cellular energy homeostasis [16]. Activation of the AMPK pathway alleviates liver damage, suppresses hepatic lipogenesis by inhibiting key lipogenic transcription factors such as SREBP1c and ChREBP, and attenuates hepatic inflammation via blunting NF-κB-mediated pro-inflammatory signaling; it also enhances mitophagy to clear damaged mitochondria and reduce oxidative stress, collectively mitigating alcoholic liver disease (ALD) in mice [17]. Intriguingly, AMPK activation inhibits ferroptosis via direct phosphorylation of ACC to curtail lipid synthesis, activation of ULK1 to promote mitophagy, and upregulation of NRF2-driven antioxidant genes, including GPX4 [18,19]. Additionally, by preventing ferroptosis, AMPK activation can lessen cardiomyocyte injury caused by ischemia/reperfusion [20]. Thus, it was hypothesized that maintaining gut flora homeostasis and focusing on the AMPK–ferroptosis axis may be a viable approach to ameliorating acute hepatic injury induced by AAI.

The clinical management of alcohol-related conditions differs substantially depending on the specific disorder. For alcohol use disorder, pharmacological agents such as naltrexone, disulfiram, and acamprosate, combined with nutritional support, represent first-line therapy options [21]. In contrast, glucocorticoids are reserved for a selected subset of patients with severe alcohol-associated hepatitis and are not routinely used for alcohol use disorder or acute intoxication [22]. Conversely, natural products show promise for treating acute alcohol intoxication. There is increasing interest in the capacity of several dietary ingredients as supplementary therapies since, in contrast to countless synthetic drugs, they have been used by humans for many decades, and the nutritional doses show good tolerance [23]. Here, an Alcohol Relief Formula (ARF) was developed that contained *Lactiplantibacillus plantarum* LTJ30, *Pediococcus acidilactici* LTJ28, Gouqi (*Lycium barbarum*) extract, Gegen (*Pueraria lobata* root) extract, soybean peptide, and Cili (*Rosa roxburghii Tratt*) extract, to reduce the discomfort caused by acute alcohol intoxication. According to our anticipated design, among the above components, lactic acid bacteria and soybean peptide should exert protective effects on the intestinal mucosal barrier and mitigate intestinal inflammation [24,25,26]. Gegen extract has been demonstrated to attenuate alcoholic liver disease by regulating ethanol metabolism [27]. Cili extract has been shown to reduce inflammation and improve lipid metabolism in mice [28]. Gouqi has been recognized in traditional Chinese medicine for its excellent liver-protecting effects for thousands of years, and it has also been shown to alleviate anxiety disorders by regulating oxidative stress and ferroptosis [29]. Additionally, all the components could modulate the gut microbiota in the role of probiotics and prebiotics. Furthermore, our previous study has demonstrated that *P. acidilactici* LTJ28 and *L. plantarum* LTJ30, which were both isolated from the fermented grains of Chinese baijiu, afford protection against alcoholic liver injury by enhancing lipid metabolism and attenuating oxidative stress [30]. In the present study, the efficacy of this combined formula was investigated.

## 2. Materials and Methods

### 2.1. Reagents and Materials

Baijiu (56% Alcohol by Volume (ABV)) was bought from Beijing Red Star Co., Ltd. (Beijing, China). *P. acidilactici* LTJ28 and *L. plantarum* LTJ30 were isolated from the fermented grains of soy sauce aroma type baijiu in North China and conserved in the General Microbiology Centre of China Microbial Strain Conservation and Management Committee (CGMCC) with the strain conservation numbers CGMCC NO. 20087 and CGMCC NO. 25371, respectively. Gegen extract, Gouqi extract, and Cili extract were supplied by Nanjing Herb-Source Bio-Technology Co., Ltd. (Nanjing, China). Soybean peptide was obtained from Shanghai Macklin Biochemical Co., Ltd. (Shanghai, China). Silymarin was provided by Zhejiang Jinhua CONBA Bio-pharm. Co., Ltd. (Jinhua, China). Nanjing Jiancheng Bioengineering Institute (Nanjing, China) provided kits for blood alcohol concentration (BAC), ADH, alanine aminotransferase (ALT), alkaline phosphatase (ALP), aspartate aminotransferase (AST), GSH, and malondialdehyde (MDA). Superoxide dismutase (SOD) was obtained from Solarbio Science and Technology Co., Ltd. (Beijing, China). ALDH, interleukin-6 (IL-6), and interleukin-1β (IL-1β) ELISA kits were purchased from Zhuocai Biotechnology Co., Ltd. (Shanghai, China) with catalog numbers ZC-56477, ZC-37988, and ZC-37974, respectively.

### 2.2. Design of Animal Experiment

Six-week-old specific pathogen-free (SPF) grade male Kunming mice (20 ± 2 g), free of designated pathogenic microorganisms and parasites, were obtained from SPF Biotechnology Co., Ltd. (Beijing, China; license SCXK[Jin] 2019-0004). The animal study protocol was approved by the Animal Care and Ethics Committee of Tianjin University of Science and Technology (approval number SWXY-20240114174). All experimental procedures were conducted per the guidelines in the Guide for the Care and Use of Laboratory Animals, and all efforts were made to minimize animal suffering.

Based on the group sizes commonly used in acute alcohol-induced liver injury studies, which have been shown to provide adequate statistical power, ninety-nine mice were randomly assigned to nine groups (*n* = 11 per group) using a computer-generated random number sequence. Experimenters were blinded to group allocation during the intervention and sample collection phases, and blinding was maintained until all analyses were completed.

Ninety-nine Kunming mice were enrolled into nine groups: a normal control group (NC); a drunk model group (M); a positive control group treated with 0.1248 g/kg silymarin (PC); a probiotic group treated with 5 × 10^10^ CFU/kg *P. acidilactici* LTJ28 and 5 × 10^10^ CFU/kg *L. plantarum* LTJ30 (L); four probiotic combination groups treated with the same dose of probiotics plus 1.25 g/kg Gegen extract (PL), 0.6 g/kg Cili extract (RL), 1.2 g/kg soybean peptide (SPL), or 0.4 g/kg Gouqi extract (LbL), respectively; and the full formula group (ARF) treated with the above probiotics, 1.25 g/kg Gegen extract, 0.4 g/kg Gouqi extract, 0.6 g/kg Cili extract and 1.2 g/kg soybean peptide.

During the experiment, one or two mice died across groups due to acute ethanol gavage-related complications, primarily aspiration or excessive central nervous system depression. These losses were unrelated to any specific intervention and occurred randomly. Affected animals were excluded from all analyses, and the final numbers of surviving animals per group (9–10) are indicated in Table 1 and the corresponding figure legends.

**Figure 1:**
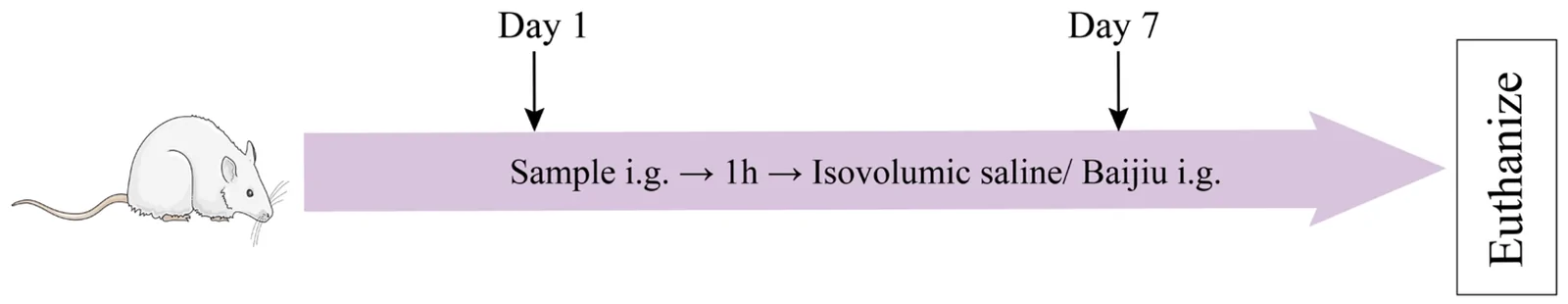
Schematic overview of the experimental design and study workflow. Kunming mice received two cycles of high-dose ethanol gavage (13 mL/kg, 56% ABV, i.g.) on days 1 and 7, with test samples administered 1 h prior to each ethanol challenge. The NC group received isovolumetric saline via gavage at both the sample and alcohol administration time points. Groups: M (saline), PC (silymarin), L (LTJ28 and LTJ30), PL (LTJ28, LTJ30, and Gegen extract), RL (LTJ28, LTJ30, and Cili extract), SPL (LTJ28, LTJ30, and soybean peptide), LbL (LTJ28, LTJ30, and Gouqi extract), and ARF (LTJ28, LTJ30, Gegen extract, Gouqi extract, Cili extract, and soybean peptide). Liver and blood were harvested for subsequent analyses. Abbreviations: i.g., intragastric gavage.

The schedule of the experimental protocol and sample administration is shown in Figure 1. Before the experiment, the mice were fasted for 12 h, and then the NC group, M group, PC group, L group, PL group, RL group, SPL group, LbL group, and the ARF groups were given sterile saline, positive drug silymarin, and different samples for each group by gavage at a dose of 5 mL/kg, respectively. Then, 1 h later, 56% ABV baijiu at a dose of 13 mL/kg was administered, except for the NC group. In the NC group, sterile saline was used instead of baijiu.

### 2.3. Assessment of the Drunken and Sober Time

After alcohol gavage in mice, drunkenness was indicated by the disappearance of the righting reflex, unsteady crawling, hind belly dragging, and rolling over. Sobriety was indicated by the recovery of the righting reflex, free movement, dexterity, alertness, and smooth gait. By monitoring behavioral responses and recording the number of drunk and dead mice, along with the times of alcohol administration, loss of the righting reflex, and recovery, we calculated drunken rate, intoxication latency, sleep time, and sobriety time.

Specifically:

Drunken rate = Number of drunk mice/Number of live mice × 100%

Latency period to drunkenness = Righting reflex disappearance time − Alcohol administration time

Sleep time = Righting reflex recovery time − Righting reflex disappearance time

Sobriety time = Righting reflex recovery time − Alcohol administration time

### 2.4. Biochemical Analysis

Venous blood from euthanized mice was centrifuged at 3500 rpm for 10 min. The resulting serum (upper layer) was collected for analysis. For homogenization, 0.05 g of liver tissue was precisely weighed and placed in 500 μL of buffer. The levels of serum BAC, ALT, AST, ALP, SOD, GSH, NO, and liver ADH and MDA were determined spectrophotometrically using commercially available kits from Nanjing Jiancheng Bioengineering Institute. Liver ALDH, IL-6, and IL-1β levels were measured by the ELISA kit following the manufacturer’s instructions. Biochemical assays were performed using samples from nine mice per group.

### 2.5. Histopathological Analysis

Liver tissue samples were fixed, paraffin-embedded, sectioned, and stained with hematoxylin and eosin (H&E). Histopathological evaluation was performed in a blinded manner using light microscopy. Liver injury was evaluated using a 0–4 grading system [31], with scores independently assigned by three blinded reviewers. Histopathological scoring of liver sections was performed independently by three investigators blinded to group allocation. Discrepancies were resolved through mutual consultation.

### 2.6. Quantitative Real-Time Polymerase Chain Reaction

After mice were euthanized, liver tissue was harvested and immediately stored at −80 °C for subsequent qRT-PCR analysis. Quantitative real-time PCR was performed following key MIQE recommendations. Primer amplification efficiencies were determined, and melt-curve analysis was carried out to verify specific amplification for each target gene. Only primers with acceptable amplification efficiency were adopted for subsequent quantification. Total RNA was extracted from liver tissue samples using TRIzol reagent, with six mice randomly selected from each of the nine groups for analysis (Thermo Fisher Scientific, Waltham, MA, USA). cDNA was synthesized following the manufacturer’s instructions (TransGen Biotech Co., Ltd., Beijing, China). Quantitative real-time PCR was performed with a Thermo Fisher Scientific QuantStudio 1 Plus system using SYBR Green qPCR Master Mix (TransGen Biotech Co., Ltd., Beijing, China). All experiments were conducted in triplicate. The relative gene transcription levels were calculated using the 2^−ΔΔCT^ method, with gene expression levels normalized to glyceraldehyde-3-phosphate dehydrogenase (Gapdh). Primer sequences used for mouse targets are listed in Table 2, and all primers were designed using NCBI Primer-BLAST (https://www.ncbi.nlm.nih.gov/tools/primer-blast/, accessed on 1 July 2026) based on the mouse gene sequences obtained from GenBank.

### 2.7. Western Blot Analysis

Liver tissue was homogenized using a grinder in RIPA lysis buffer containing protease and phosphatase inhibitors (Beyotime Biotechnology, Shanghai, China). Equal amounts of protein were separated via 7.5% or 12.5% SDS-PAGE gels alongside a pre-stained protein ladder (PR1910, Solarbio, Beijing, China) and transferred to nitrocellulose (NC) membranes at a constant current of 300 mA (Cytiva, Marlborough, MA, USA). The membranes were blocked using a rapid blocking buffer for 10 min, followed by incubation with specific primary antibodies against GPX4 (ET1706-45, HUABIO, Hangzhou, China), SLC7A11 (HA721868, HUABIO), AMPK (ET1608-40, HUABIO), p-AMPK (ET1612-72, HUABIO), FSP1 (EPR14639, Abcam, Cambridge, UK), and β-actin (20536-1-AP, Proteintech, Wuhan, China) for 2 h at dilutions of 1:5000 for β-actin, GPX4 and AMPK, 1:1000 for SLC7A11, and 1:500 for FSP1 and p-AMPK, all prepared in TBST. After incubation, membranes were washed three times with TBST (5 min each), then incubated with corresponding IRDye 680RD secondary antibodies (LI-COR, Lincoln, NE, USA) at a 1:10,000 dilution prepared in TBST in the dark for 1 h at room temperature. Following three additional TBST washes, immunodetection was performed using an Odyssey CLx infrared laser imaging system (LI-COR, Lincoln, NE, USA). Band intensities were quantified with ImageJ software (version 1.49v; National Institutes of Health, Bethesda, MD, USA), and target protein expression levels were normalized to β-actin.

### 2.8. Determination of SCFAs via GC

Accordingly, 0.1 g of cecal contents were homogenized with 1 mL of PBS, and the suspension was spun at 10,000 rpm for 5 min at 4 °C. A 750 µL aliquot of the supernatant was mixed with 250 µL of 15% methanol–sulfuric acid solution and subjected to gas chromatography (Agilent GC7890A, Santa Clara, CA, USA). The analysis employed an HP-5 column (30 m × 320 µm × 0.25 µm) with helium as the carrier gas at a flow rate of 2 mL/min. The inlet was held at 250 °C; 1 µL of sample was injected with a split ratio of 1:1; and the column temperature was programmed to start at 90 °C (held for 6 min) before rising to 200 °C at 10 °C/min. Detection was carried out with the heater set at 250 °C, H_2_ flow at 30 mL/min, and air flow at 300 mL/min. SCFA concentrations were calculated from a standard calibration curve.

### 2.9. Gut Microbiota Analysis

Colon content samples were collected from mice, immediately frozen at −80 °C, and transported to Hangzhou Astrocyte Technology Co., Ltd. (Hangzhou, China) for 16S rRNA gene sequencing. The procedure included genomic DNA extraction and amplification of the V3–V4 hypervariable region of the bacterial 16S rRNA gene using specific primers 341F (5′-CCTACGGGNGGCWGCAG-3′) and 785R (5′-GACTACHVGGGTATCTAATCC-3′). PCR products were purified using AMPure XP Beads and the QIAquick Gel Extraction Kit, and library quality was assessed by Qubit, Qseq100, and the KAPA Library Quantification Kit. High-throughput sequencing was performed on the Illumina NovaSeq 6000 platform with a paired-end 250 bp read length, and amplicon sequence variants (ASVs) were analyzed using the DADA2 pipeline. Alpha diversity differences among groups were assessed using the Wilcoxon rank-sum test and Kruskal–Wallis H test. Beta diversity significance was evaluated using ADONIS, Anosim, MRPP, and Amova. Differential taxa were identified using LEfSe analysis; no additional multiple-testing correction was applied to LEfSe results, and these findings should therefore be considered exploratory.

### 2.10. Statistical Analysis

Statistical analysis of behavioral, physiological, and biochemical data was performed using GraphPad Prism version 10.4.1 software. Data are presented as mean ± standard deviation (SD). Normality was assessed using the Shapiro–Wilk test, and homogeneity of variances was confirmed by Levene’s test before analysis. One-way analysis of variance (ANOVA) followed by Dunnett’s multiple comparison test was used to identify differences, with a probability (P) value less than 0.05 deemed statistically significant. For Pearson’s correlation analyses between SCFAs and hepatic parameters, multiple-testing correction was applied. During administration and sample collection, the experimenters were blinded to group allocation to minimize bias. Predefined humane endpoints, including criteria for early euthanasia, were established according to institutional animal care guidelines and were not triggered during the study.

## 3. Results

### 3.1. ARF Promotes the Recovery of Sobriety in Acutely Intoxicated Mice

To evaluate the anti-drunkenness efficacy of individual components within the ARF, the loss of righting reflex (LORR) assay was employed to determine the latency to drunkenness and recovery time in mice [32]. As shown in Table 1, compared with the model (M) group, the incidence of drunkenness decreased from 100.0% to 90.00%, 77.78%, 80.00%, 66.7%, 77.78%, and 60.00% in the L, PL, LbL, RL, SPL, and ARF groups, respectively. Regarding the preventive hepatoprotective effects on drunkenness and recovery, ARF demonstrated significant benefits. While alcohol administration alone led to a latency period to drunkenness of 10.6 min, ARF and the other corresponding ingredient combination groups prolonged this latency period to 15–39 min, with the highest value being nearly four times that of the M group. Conversely, sleep duration was substantially decreased in the six tested formulation groups. The duration was reduced from 330.60 min (M group) to between 126.67 min and 149.17 min (RL, PL, ARF, and LbL groups).

The sobriety time was decreased by both the L and PC group pretreatments. In comparison to lactic acid bacteria alone, probiotics together with Cili extract, Gouqi extract, Gegen extract, and soybean peptide lessened the sobriety period. With all the assessed components included, the ARF group performed better than the PC group, decreasing the model group’s sobriety period from 341.20 min to 134.00 min. These findings indicated that lactic acid bacteria are effective in accelerating recovery from intoxication; this is further enhanced when combined with Gegen extract, Gouqi extract, soybean peptide, and Cili extract. Specifically, the LbL group was more effective in reducing sleep time. However, the SPL group was better at preventing drunkenness. Both the PL and RL groups were effective for both sobering up and preventing drunkenness. The ARF group used all the components together. This combination gave the most comprehensive protective profile of all the groups.

### 3.2. ARF Attenuates Ethanol-Induced Oxidative Stress and Hepatic Inflammation

**Figure 2:**
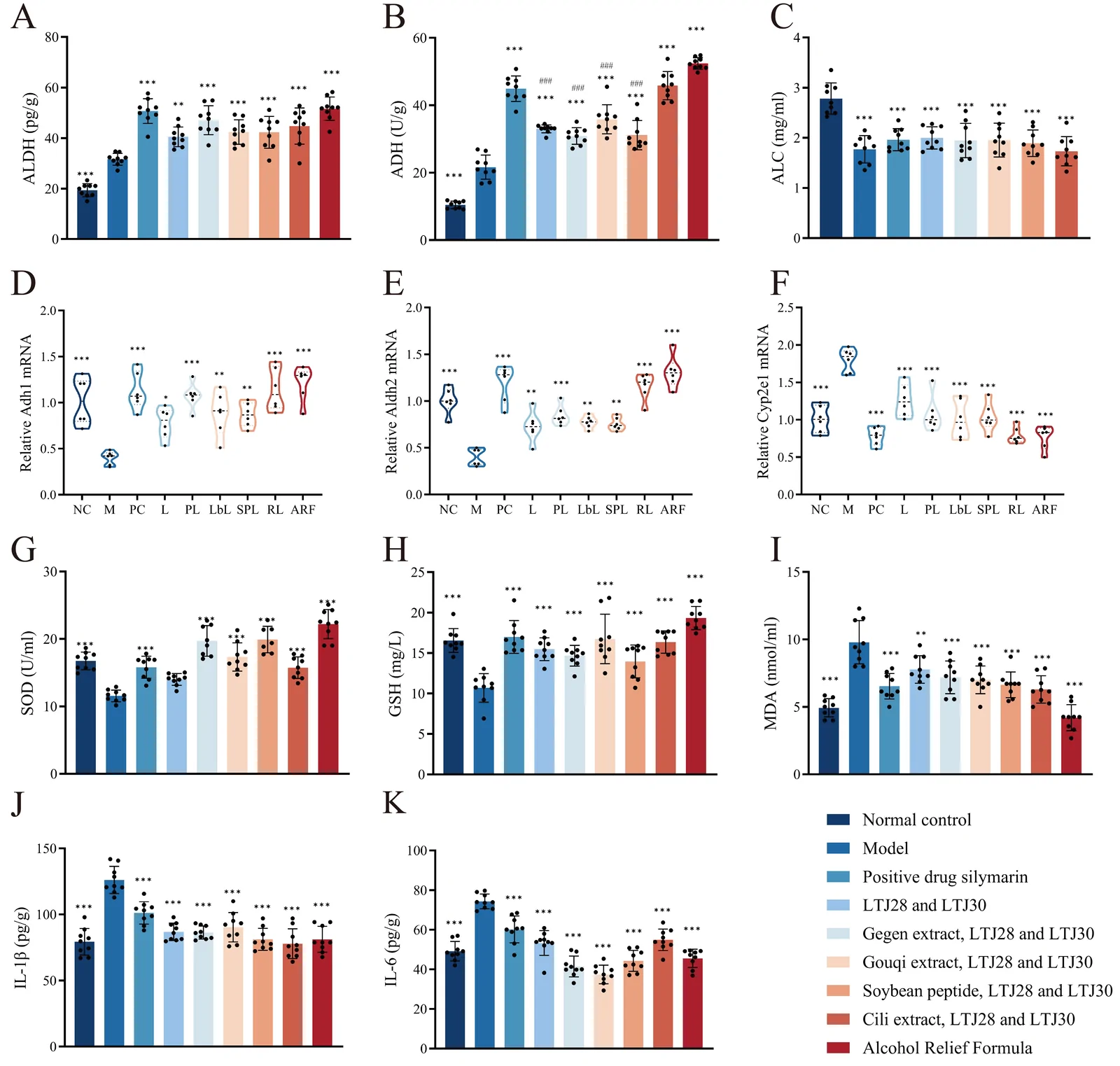
ARF enhances ethanol metabolism and attenuates oxidative stress and inflammation in mice with acute alcoholic liver injury. (**A**) Liver ALDH activity. (**B**) Liver ADH activity. (**C**) Serum ALC levels. (**D**) Liver ADH mRNA levels. (**E**) Liver ALDH mRNA levels. (**F**) Liver CYP2E1 mRNA levels. (**G**) Serum SOD levels. (**H**) Serum GSH levels. (**I**) Serum MDA levels. (**J**) Liver IL-1β levels. (**K**) Liver IL-6 levels. Data are presented as the mean ± SD. For serum and tissue biochemical assays, *n* = 9 biological replicates; for mRNA measurements, *n* = 6 biological replicates. * *p* < 0.05, ** *p* < 0.01, *** *p* < 0.001 vs. the M group. ### *p* < 0.001 compared to the model group. Abbreviations: NC, normal control; M, alcohol model; PC, positive control (silymarin); L, probiotics (*P. acidilactic* LTJ28 and *L. plantarum* LTJ30); PL, L + Gegen extract; SPL, L + soybean peptide; LbL, L + Gouqi extract; RL, L + Cili extract; ARF, L + Gegen extract + Gouqi extract + Cili extract + soybean peptide.

In the setting of AAI, the duration of intoxication and recovery in mice displaying a loss of righting reflex serves as the most direct phenotypic indicator of intoxication. Meanwhile, biochemical markers of alcohol metabolism provide substantial evidence of the organism’s response to alcohol exposure. As shown in Figure 2A,B, liver ADH activity increased from 10.46 to 21.64 U/g and ALDH activity from 19.42 to 31.65 pg/g in the model group compared with normal controls, confirming the successful induction of acute ethanol intoxication. Pretreatment with silymarin or any of the tested formulations was associated with further increased activities of both enzymes. Remarkably, in this respect, the mentioned six formulations outperformed the positive control (PC). Probiotics and the Cili extract were noticeably more successful at raising ADH activity than the other natural combinations. At 2.78 mg·mL^−1^, the model (M) group showed peak serum ethanol levels 2.5 h after administering alcohol (Figure 2C). Blood alcohol concentrations were lowered to 1.77–2.00 mg·mL^−1^ when silymarin or any of the evaluated formulations was used as pretreatment. To further elucidate the mechanistic basis of formulations that enhanced ethanol clearance, we quantified hepatic mRNA levels of key ethanol-metabolizing enzymes. Acute alcohol exposure markedly downregulated the transcription of alcohol dehydrogenase 1 (*Adh1*) and aldehyde dehydrogenase 2 (*Aldh2*), while inducing Cyp2e1 overexpression (Figure 2D–F). All the tested formulations were associated with recovered Adh1 and Aldh2 expression and lower alcohol-induced Cyp2e1 upregulation.

Ethanol consumption disrupts the hepatic redox balance, promoting free radical generation, oxidative stress, metabolic dysfunction, and lipid peroxidation [33]. Therefore, alcohol-induced oxidative stress was examined. The results showed that mice in the drunk model group exhibited a significant decrease in serum SOD and GSH levels, along with a notable increase in MDA (Figure 2G–I), while all the tested formulations were associated with partially recovered SOD and GSH concentrations and reduced MDA accumulation, indicating attenuation of alcohol-induced oxidative imbalance in this preventive model. Furthermore, the pro-inflammatory cytokines IL-6 and IL-1β in liver homogenates were quantified. As shown in Figure 2J,K, acute alcohol consumption significantly elevated both cytokines, whereas silymarin and all six ACF-based formulations markedly attenuated these increases, consistent with observations in established alcoholic liver disease models. According to earlier studies on the anti-inflammatory qualities of goji berry extract, the LbL group showed the greatest reduction in IL-6 activity [34].

### 3.3. ARF Alleviates Acute Alcohol-Induced Liver Injury

**Figure 3:**
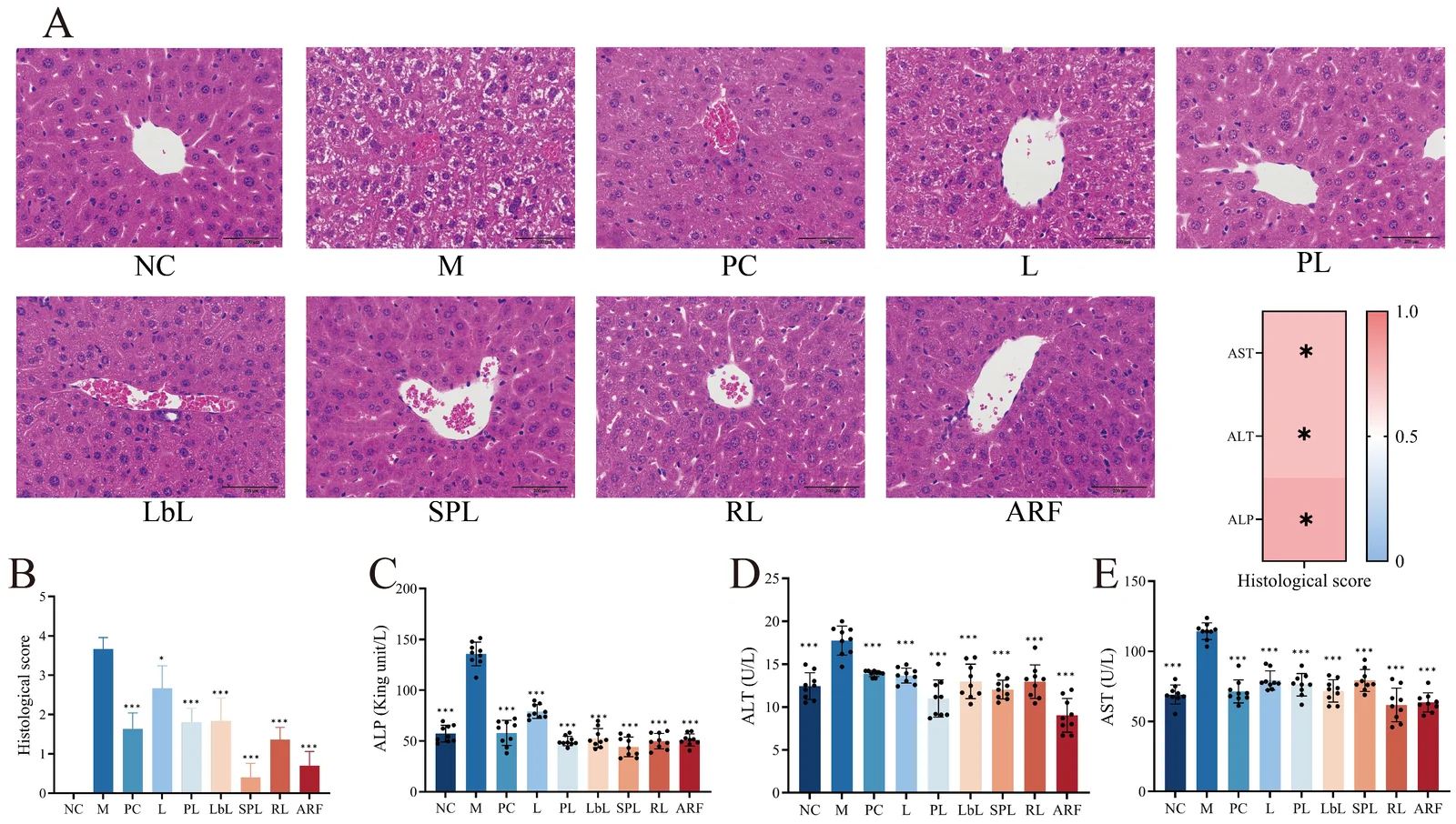
Hepatic injury caused by alcohol is lessened by ARF. (**A**) Liver histology after H&E staining (400×); correlation analysis between serum AST, ALT, and ALP levels relative to histological scores. (**B**) Histological score for liver sections. (**C**) Levels of serum ALP. (**D**) Levels of serum ALT. (**E**) Levels of serum AST. Mean ± SD represents all data. For serum biochemical assays, *n* = 9 biological replicates; for histological scoring, *n* = 3 biological replicates. Scale bars, 200 μm. * *p* < 0.05, and *** *p* < 0.001 vs. the M group.

Then, the hepatoprotective impact of each ARF component was assessed using H&E staining. Healthy control animals’ liver tissues showed no signs of severe degeneration or apoptosis, with an undamaged lobular structure, distinct sinusoids, and uniformly arranged hepatocytes with conspicuous nuclei. In contrast, animals exposed to ethanol displayed enlarged hepatocytes with noticeable cytoplasmic vacuolization along with altered lobular architecture, which is in line with hepatocellular damage attributed to alcohol. In all examined groups, these pathogenic changes were substantially attenuated following ARF pretreatment. Particularly, the ARF and PL groups showed the greatest histoarchitectural improvement, and the cellular morphology resembled that of healthy controls (Figure 3A).

Hepatocyte injury was quantified by measuring ALT, ALP, and AST levels in the serum. When the hepatocyte canalicular membrane is damaged, the glycoprotein ALP enters the bloodstream. Sensitive hepatocellular damage indicators, ALT and AST, enter the serum when cell membrane integrity is compromised [35]. Mice in the model group showed significantly increased ALT, ALP, and AST activities compared to controls after 2.5 h of receiving 56% ethanol (*p* < 0.01). Thus, acute alcoholic liver injury was successfully induced. The serum levels of these three enzymes were substantially lower in all six formulation groups. In contrast to probiotics treated with any one natural product, ARF was associated with the strongest attenuation of ALT rise, suggesting that the combined effect of probiotics with various botanicals correlates with greater hepatoprotection in this preventive setting.

### 3.4. By Activating the AMPK Pathway, ARF Intervention Is Accompanied by Reduced Hepatic Ferroptosis

**Figure 4:**
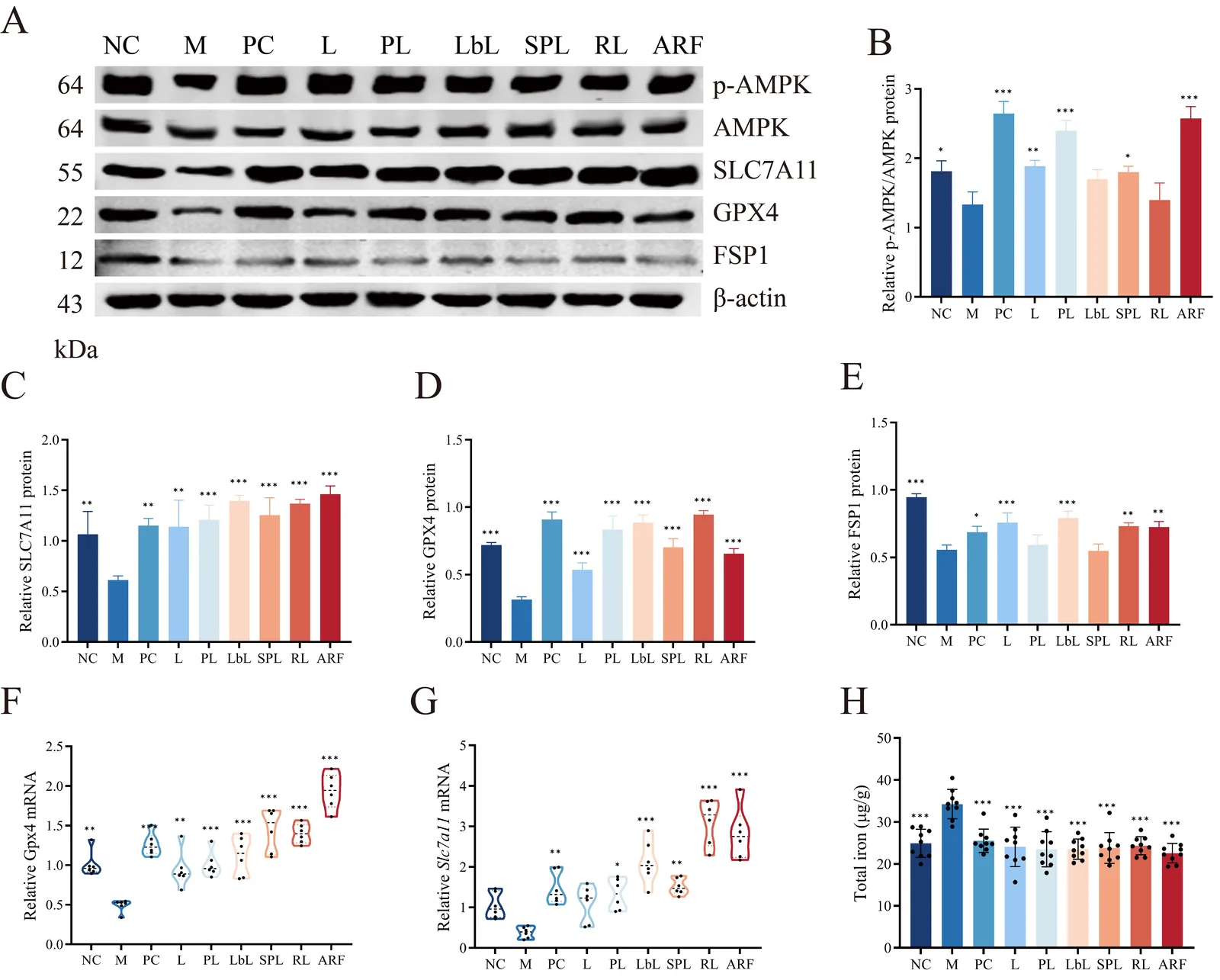
By triggering the AMPK pathway, ARF reduces ferroptosis in acute alcoholic liver injury. (**A**–**E**) Proteins related to the AMPK pathway (AMPK and p-AMPK) and ferroptosis (GPX4, SLC7A11, and FSP1) in the liver. (**F**) mRNA levels of *Gpx4*. (**G**) mRNA levels of *Slc7a11*. (**H**) Liver total iron levels. Mean ± SD represents all data. For Western blotting, *n* = 3 biological replicates; for mRNA measurements, *n* = 6 biological replicates. * *p* < 0.05, ** *p* < 0.01, and *** *p* < 0.001 vs. the M group.

Next, we assessed the AMPK phosphorylation status. Binge alcohol intake considerably suppressed AMPK phosphorylation (Figure 4A,B), whereas ARF pretreatment was associated with elevated phospho-AMPK levels. Compared with controls, the model group showed a downregulation of both solute carrier family 7 member 11 (SLC7A11) and GPX4 protein expression, as evidenced by immunoblotting analysis (Figure 4A,C,D). These changes are consistent with the function of AMPK in ferroptosis control. Notably, this alcohol-induced suppression was attenuated by ARF pretreatment. Additionally, although the efficacy of each combination varied, the six formulations were associated with maintained FSP1 expression in the context of ethanol-driven downregulation (Figure 4A,E).

Alcohol-suppressed mRNA levels of *Gpx4* and *Slc7a11* in the liver showed recovery towards control levels following ARF pretreatment, according to quantitative real-time PCR (Figure 4F,G). In particular, *Gpx4* mRNA levels in the LbL, SPL, ARF, and RL groups rose to 193.40%, 127.15%, 228.15%, and 131.24%, respectively. Compared to the model group, mRNA expression rose to 349.37%, 240.59%, 289.24%, 400.00%, 359.45%, 216.85%, and 472.39% for Slc7a11, silymarin, and all formulation pretreatment groups (L, PL, LbL, RL, SPL, and ARF), respectively. Total iron, another important marker of iron-related mortality, was also found to be significantly reduced in the livers of all intervention groups (Figure 4H). Together, these observations indicate that ARF pretreatment is associated with AMPK signaling modulation, elevated GPX4, SLC7A11, and FSP1 expression, and attenuation of ferroptosis-related changes, which correlate with mitigated alcohol-induced liver injury in this preventive model.

### 3.5. ARF Modifies the Gut Flora Composition in Mice

**Figure 5:**
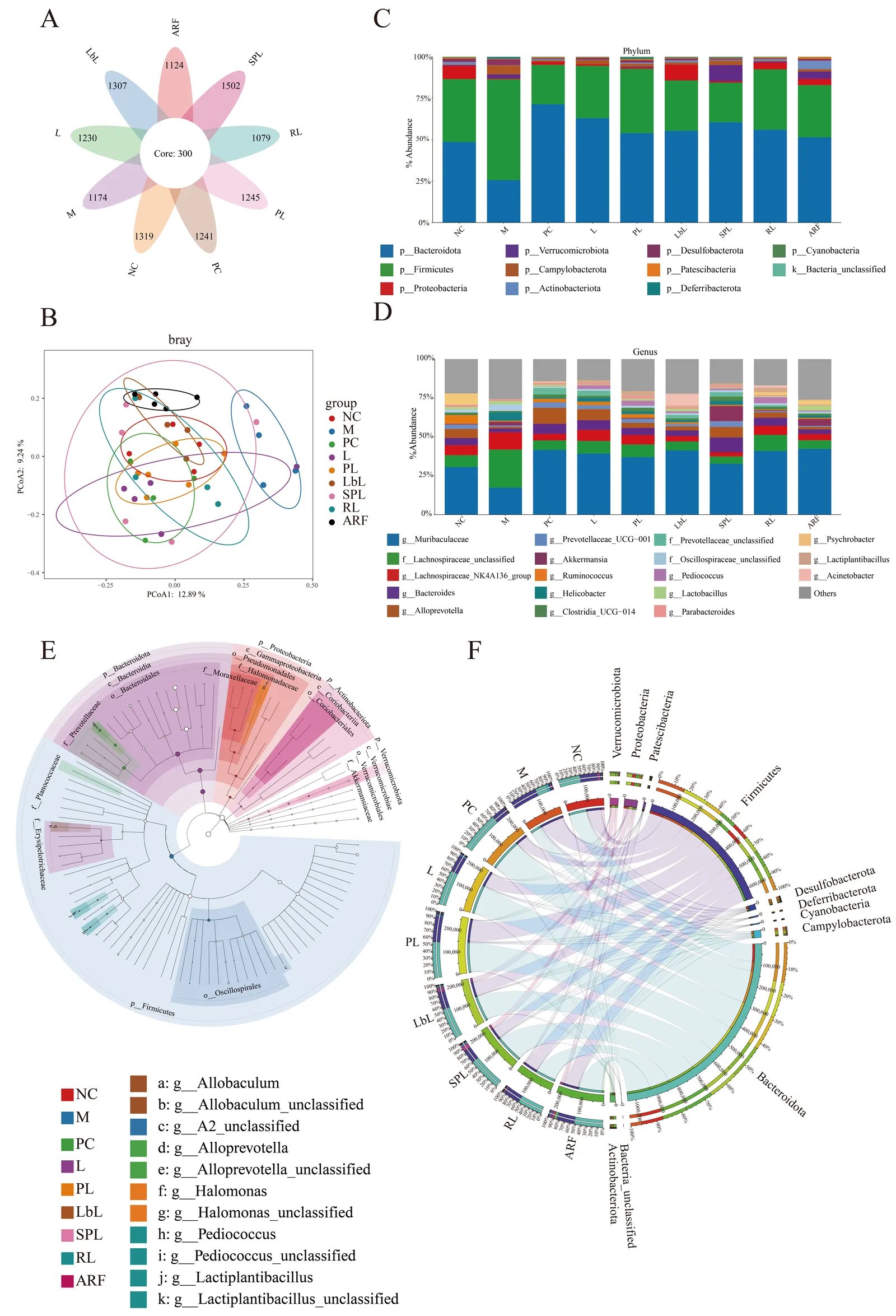
ARF restores gut flora dysbiosis triggered by alcohol. Colon contents are used to examine the composition of the gut flora. (**A**) Venn diagram showing operational taxonomic units. (**B**) Principal coordinate analysis of β-diversity. (**C**,**D**) Composition of (**C**) phylum- and (**D**) genus-level microbial communities. (**E**) LEfSe analysis showing taxa with substantial variation in abundance. (**F**) Circos plot showing the relationship between the leading 10 genera and each group.

Alcohol intake alters the gut flora composition and increases the permeability of the gut, among other features of the intestinal milieu [36]. Apart from ethanol’s direct hepatotoxicity, accumulating intestinal disruptions triggered by alcohol exacerbate the development of liver injury. The 16S rRNA (V3–V4) sequencing of colonic contents was conducted to investigate how certain ARF components were associated with gut flora changes after acute alcoholic liver injury. The rarefaction curve showed that the sequencing depth for the nine groups was sufficient to cover all taxonomic groups (Supplementary Figure S1). Bacterial community analysis indicated that each studied formulation was associated with attenuation of alcohol-triggered dysbiosis. As shown in Supplemental Table S1, the Chao, Ace, and Shannon indices were decreased in the M group compared with the NC group, whereas the InvSimpson index showed a numerical increase in the model group. These indices were partially recovered to varying degrees following intervention with groups L, PL, LbL, RL, SPL, and ARF. Operational taxonomic unit distribution across groups is depicted in the petal plot (Figure 5A), and the normal control (NC) and model (M) groups are distinguished by principal coordinate analysis (PCoA). Particularly, the PL, PC, RL, LbL, and complete ARF groups generated unique clusters that were entirely disconnected from the M group and more closely aligned with the NC group (Figure 5B). Therefore, in mice with alcoholic liver injury, ARF was associated with a more normalized dysbiotic composition and altered the gut flora system.

Alcohol intake decreased the phylum-level relative abundance of *Campylobacterota*, *Bacteroidetes*, *Deferribacterota*, and *Desulfobacterota*. ARF pretreatment was associated with attenuating these changes (Figure 5C). Different intervention-related changes in gut flora composition were identified by additional genus-level profiling (Figure 5D). Notably, after receiving RL and LbL pretreatment, Helicobacter was noticeably enriched in the M group, but its abundance returned to levels similar to the NC group. Conversely, *Muribaculaceae* increased in all intervention groups; *Akkermansia* was more abundant in the SPL group, and *Alloprevotella* was specifically promoted by the L group. The complete ARF formulation produced the most functionally cohesive and structurally coordinated microbial environment by concurrently showing increased abundance of all favorable genera.

Important bacterial taxa related to each intervention group were detected by LEfSe analysis, and dendrogram analysis confirmed these results (Figure 5E,F). In the L group, *Bacteroidales* and *Erysipelatoclostridiaceae* were significantly enriched. In the PL group, *Halomonas* and *Rhizobiaceae* were significantly enriched. In the LbL group, *Allobaculum*, Rhizobiales, and *Devosiaceae* were significantly enriched. In the SPL group, *Akkermansiaceae* were significantly enriched. In the RL group, *Pediococcus* and *Lactiplantibacillus* were significantly enriched. In the ARF group, Actinobacteria were significantly enriched. The 16S analysis results showed that alcohol intake was associated with metabolic impairment and microbial dysbiosis in mice and disruption of the anaerobic gut environment. Despite only attenuating these changes, *P. acidilactic* LTJ28 and *L. plantarum* LTJ30 pretreatment showed positive associations with the gut flora. In contrast, these probiotic combinations (ARFs) with soybean peptide, Gouqi extract, Gegen extract, and Cili extract were associated with more pronounced gut flora architecture modulation and greater homeostatic recovery, highlighting the benefits of this integrated nutritional approach.

### 3.6. ARF Restores SCFA Levels in the Intestines of Mice

**Figure 6:**
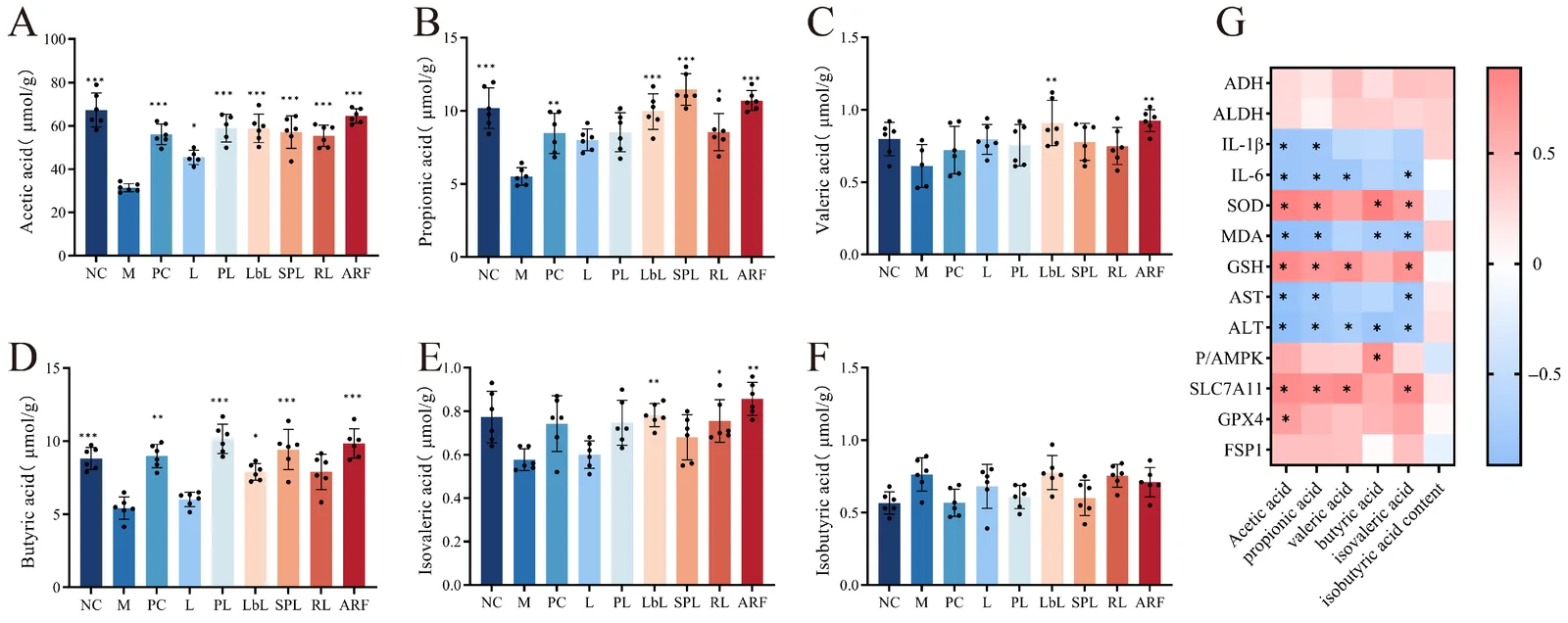
ARF restores short-chain fatty acid depletion in mice caused by alcohol. (**A**–**F**) Cecal concentrations of SCFAs, including (**A**) acetic acid, (**B**) propionic acid, (**C**) valeric acid, (**D**) butyric acid, (**E**) isovaleric acid, and (**F**) isobutyric acid. (**G**) Correlation analysis between SCFAs and parameters related to ferroptosis or hepatic damage markers. All measurements used *n* = 6 biological replicates. Significance is shown as * *p* < 0.05, ** *p* < 0.01, and *** *p* < 0.001 in comparison to the model group.

Subsequently, we measured the SCFA levels in the cecum. Following acute alcohol exposure, the model (M) group exhibited significant reductions in cecal acetate, propionate, and butyrate by 50.7% (*p* < 0.001), 52.4% (*p* < 0.01), and 34.7% (*p* < 0.05), respectively (Figure 6A–F). Valerate and isovalerate levels were also decreased (−21.7% and −21.5%, respectively), whereas isobutyrate increased by 32.5%. These changes observed in the model group were attenuated by all the mentioned formulations in this preventive setting. Probiotics in combination with botanical compounds (LbL, PL, RL, SPL, and ARF) brought SCFA profiles back toward normal, compared with probiotics alone (L group). With butyrate and acetate rising by 71.5% and 111.7%, respectively, robust restoration was observed in the ARF group compared with the model group (*p* < 0.001). These values were not significantly different from the control group values.

Pearson’s correlation analysis was used to elaborate on the functional association between SCFAs in the intestine and liver injury caused by alcohol (Figure 6G). The levels of propionate, acetate, and butyrate were negatively related to MDA and ALT levels (*p* < 0.05) and positively related to SOD activity (*p* < 0.05), suggesting enhanced redox balance and lower hepatocellular injury. Additionally, there was an inverse correlation (*p* < 0.05) between propionate and acetate and IL-6 and IL-1β. Propionate, acetate, and valerate levels were positively related to SLC7A11 expression (*p* < 0.05), and butyrate levels were positively related to AMPK phosphorylation (*p* < 0.05), as shown by protein expression analysis.

## 4. Discussion

Acute alcohol intoxication is driven by the rapid accumulation of ethanol coupled with inadequate metabolic clearance. Excessive alcohol consumption damages liver tissue, disrupts liver metabolism, and is accompanied by gut microbiota dysbiosis [37]. This study investigated Alcohol Relief Formula (a formula containing *L*. *plantarum* LTJ30, *P*. *acidilactici* LTJ28, Gouqi extract, Gegen extract, soybean peptide, and Cili extract) on the latency period to drunkenness, the sleep time, and the sobriety time in AAI mice. Our results indicated that lactic acid bacteria alone made a difference; their real strength emerged when combined with botanical extracts and soybean peptides. LbL significantly shortened sleep time and accelerated recovery, demonstrating a pronounced sobering effect. SPL had a significant effect on prolonging the latency period to drunkenness, with no obvious difference from group L in terms of reducing sleep time. PL and RL not only prolonged the latency period to drunkenness but also shortened the total sobriety time. ARF, by uniting all its components, simultaneously prolonged the latency to drunkenness, shortened sleep time, and accelerated recovery.

In the liver, ethanol is oxidized to acetaldehyde by ADH and then to acetate by ALDH, which enters the TCA cycle [38,39]. ADH and ALDH activities are widely used as indicators of ethanol metabolism [40]. In this study, acute alcohol intake elevated hepatic ADH and ALDH levels, but ALDH activity remained insufficient to prevent acetaldehyde-induced damage. ARF enhanced ethanol clearance by restoring both enzymes. Notably, Cili extract combined with probiotics was most effective at raising ADH activity, while Gegen extract with probiotics most potently boosted ALDH activity. CYP2E1 also contributes to ethanol metabolism, generating ROS and oxidative stress [41], and acetaldehyde drives inflammation via IL-1β and IL-6 [42]. Early ARF intervention protected against liver damage by suppressing oxidative stress and inflammation. Although kudzu root is traditionally valued for alcohol metabolism and liver protection [27], Gegen extract paired with probiotics elevated ADH to a lesser extent while raising ALDH more effectively, thereby reducing acetaldehyde accumulation. However, the results showed that compared to other ARF components, Gegen extract paired with probiotics elevates ADH activity to a lesser extent while raising ALDH activity more effectively, thereby reducing acetaldehyde production. This mechanism is associated with its sobering effects and liver protection, though its anti-inflammatory and anti-oxidative stress activities remain limited.

Oxidative stress, iron overload, and severe lipid peroxidation are the hallmarks of ferroptosis, a type of controlled cell death that depends on iron concentrations [43]. Hepatic iron buildup worsens oxidative damage in alcoholic liver injury, augmenting death of ferroptotic hepatocytes and accelerating the course of the illness [44]. ROS produced by ethanol metabolism causes phospholipids to undergo lipid peroxidation and concurrently oxidizes both GSH and NAD(P)H, which impairs GPX4 function and disrupts the reductive pathway that transforms PLOOH to PLOH [45]. Eventually, ferroptosis is triggered by this cascade’s combined amplification of lipid peroxidation. By lowering coenzyme Q10 (CoQ), ferroptosis suppressor protein 1 (FSP1) inhibits this process, preventing the accumulation of lipid peroxide and creating an antioxidant system parallel to GPX4 [46]. Moreover, ferroptosis is regulated by AMPK, a key modulator of cellular energy homeostasis, through distinct downstream targets [47,48]. Our study further demonstrated significant downregulation of AMPK phosphorylation and pronounced ferroptotic changes in AAI model mice. Although the mechanisms by which herbs, peptides, or probiotics mediate ferroptosis under alcohol exposure remain unclear, our study showed that pretreatment with a combination of these components effectively attenuated ferroptosis-associated changes induced by acute alcohol intake. This study demonstrated that all intervention groups increased SLC7A11 protein expression in the liver. Meanwhile, LbL and RL proved more effective at enhancing GPX4 protein expression, whereas the SPL group attenuated ferroptosis-related markers primarily by promoting FSP1 expression. Interestingly, PL induced the highest AMPK phosphorylation among the six formulations excluding ARF, yet it did not stand out in protecting against ferroptosis-related changes under alcohol exposure. This suggests that ARF components may be associated with the regulation of ferroptosis-related pathways involving AMPK signaling, which may correlate with attenuation of acute alcoholic liver injury.

The gut microbiota is closely related to liver metabolism, influencing ethanol metabolism, liver inflammation, and signaling pathways such as AMPK [49]. Through 16S analysis of mouse gut microbiota, our findings revealed that *Helicobacter*, a pathobiont linked to inflamed intestines, a dysfunctional barrier, and an elevated colorectal cancer risk, was markedly enriched in the M group. After receiving RL and LbL pretreatment, *Helicobacter* abundance returned to levels comparable with those of the NC group. Furthermore, previous studies have reported that *Pediococcus*, a lactic acid-producing genus known to help regulate gut pH and inhibit pathogen growth, was decreased in alcohol-fed mice [50]. Moreover, previous studies have reported that *Akkermansia* degrades mucin, thereby strengthening the intestinal mucus barrier and promoting immune homeostasis [51]. In this study, PL pretreatment was associated with enhanced *Pediococcus*, while SPL pretreatment was associated with increased *Akkermansia* in AAI mice, as reflected in genus-level microbial communities and LEfSe analysis. We also observed that Muribaculaceae, a Bacteroidetes family previously associated with short-chain fatty acid (SCFA) production, was more abundant in all intervention groups [52]. Allobaculum, a genus reported to degrade fibers and influence SCFA release, was abundant in the LbL group; this genus has been previously associated with lower inflammation and improved intestinal integrity [44].

The liver is the major organ subjected to metabolites and microbial agents originating from the gut [53]. Intestinal homeostasis is preserved, and gut flora dysbiosis attributed to alcohol has been reported to be mitigated by SCFAs, which are important metabolites produced by microbial fermentation of undigested dietary proteins, fiber, and peptides [54]. SCFAs have been reported to provide multimodal protection in alcoholic liver injury, such as hepatic inflammation control and intestinal barrier fortification. Propionate and butyrate have been shown to work in concert to strengthen barrier integrity and reduce inflammation [55]. Specifically, propionate enhances the structure of the microbial population and helps to restore the mucus layer in the intestine. Butyrate increases the production of tight-junction proteins, which improves intestinal barrier integrity. Additionally, studies have shown that butyrate can block ferroptosis in hepatocytes by triggering AMPK-induced mitophagy [56]. In the interim, AMP is produced by the hepatic metabolism of acetate, which triggers AMPK signaling and reduces hepatocyte ferroptosis [57]. In our study, the model group exhibited a marked reduction in acetate, propionate, and butyrate levels, which were significantly restored following intervention with various components of ARF. Importantly, PL and SPL pretreatments were associated with significantly increased acetate and butyrate levels, metabolites that have been linked to the AMPK pathway. These associations suggest that elevated SCFA levels may be linked to the AMPK phosphorylation observed in mouse livers, although causality cannot be inferred from these correlational data and further validation is required.

Several limitations of this study should be acknowledged. The primary active component driving the hepatoprotective effect of ARF has not been definitively identified, as the multi-group design was primarily established to verify the combined efficacy of different components combined with probiotics, which can only preliminarily reflect the contribution trend of each ingredient without precisely quantifying its independent efficacy. In addition, only a single, pre-determined dose of ARF was tested, precluding determination of the minimum effective dose, optimal protective dose, and protective window. This study was also conducted exclusively in an acute binge alcohol exposure model, and the findings cannot be directly extrapolated to chronic alcoholic liver disease or human clinical settings. Moreover, as a preventive pretreatment study, post-exposure efficacy remains unexplored. Several translational issues—including human equivalent dose, formulation stability, long-term daily intake practicability, and regulatory compliance—have not been addressed in the current preclinical work. Further investigations using dose–response designs, component gradient titration, chronic alcohol exposure models, and post-exposure administration protocols are required to clarify the core active components, optimize the intervention regimen, and establish translational feasibility.

## 5. Conclusions

In this research, the protective impact of ARF, a nutritional formulation composed of several components, such as botanical extracts, probiotics, and soybean peptides, against acute intake of alcohol and related liver damage was thoroughly assessed in a preclinical mouse model with ARF administered prior to ethanol challenge, reflecting a preventive pretreatment design. We showed that pre-administration of ARF significantly promoted recovery from intoxication, enhanced clearance of ethanol, and reduced alcohol-triggered inflammatory responses, oxidative stress, and metabolic dysfunction in the livers of mice exposed to acute alcohol administration. Our data reveal that ARF pre-administration is associated with increased AMPK phosphorylation, upregulation of GPX4 and SLC7A11, and restoration of SCFA profiles, which together may contribute to the attenuation of acute alcohol-induced liver damage and the modulation of ferroptosis-related markers. However, the proposed gut microbiota–SCFA–AMPK–ferroptosis axis is based on correlational evidence, and causal relationships among these events remain to be determined.

In summary, our findings demonstrate that preventive ARF pretreatment exerts favorable hepatoprotective effects against acute binge-alcohol-induced liver injury in mice, and these effects are associated with coordinated changes in gut microbiota-derived metabolites and the AMPK signaling pathway related to ferroptosis. These results suggest that multi-component nutritional formulations may represent a promising preventive dietary strategy for reducing acute alcohol intoxication and related liver damage. Given the limitations of the current preclinical acute model and the preventive pretreatment design, the present findings should not be extrapolated to chronic alcohol-associated liver disease. Further validation in chronic alcohol exposure models, along with rigorous dose-optimization studies and well-designed clinical trials, is warranted to confirm the long-term efficacy, safety, and translational applicability of ARF in human populations.

## Figures and Tables

**Table 1:** Effects of ARF on sobriety recovery in acutely intoxicated mice. The asterisks represent significant differences from the M group (statistical significance: * *p* < 0.05, ** *p* < 0.01, *** *p* < 0.001).

Group	M	PC	L	PL	LbL	RL	SPL	ARF
**Number of** **live** **mice**	9	10	10	9	10	9	9	10
**Drunken rate/%**	100.00%	100.00%	90.00%	77.78%	77.78%	66.67%	80.00%	60.00%
**Latency period to drunkenness/min**	10.60 ± 3.98	21.22 ± 7.23 *	15.50 ± 5.93	43.29 ± 6.42 ***	14.50 ± 4.54	35.60 ± 10.01 ***	39.33 ± 8.71 ***	24.00 ± 9.19 *
**Sleep time/min**	330.60 ± 16.88	144.78 ± 17.13 ***	251.22 ± 59.90 **	131.86 ± 10.62 ***	149.17 ± 39.56 ***	130.40 ± 55.63 ***	213.17 ± 47.40 ***	126.67 ± 47.16 ***
**Sobriety time/min**	341.20 ± 17.77	166.00 ± 15.95 ***	270.11 ± 55.53 *	175.14 ± 12.88 **	164.70 ± 38.82 ***	166.00 ± 54.52 ***	252.50 ± 45.05 ***	134.00 ± 58.90 ***

**Table 2:** Primers and sequences for real-time qPCR.

Gene Name	Forward Primer (5′-3′)	Reverse Primer (5′-3′)	Product Size
*Cyp2e1*	CAGGAGTACAAGAACAAGGGGAT	TTTGGATGCGGGCCTCATTA	127
*Adh1*	GCACCTGGAAGGGAGCAATA	AGGACGGTACGGATGCTCTT	181
*Aldh2*	CCTGGCGTGGTCAATATCGT	TTAGGTGACCAACCTCCGTG	115
*Gpx4*	TGCCTGGATAAGTACAGGGGT	TATCGGGCATGCAGATCGAC	107
*Slc7a11*	ATCTTCGATACAAACGCCCAG	GATAATCGTCTGAACCACTTGGG	217
*Gapdh*	ATGGTGAAGGTCGGTGTGAACGG	TGGAACATGTAGACCATGTAGTTGAGG	137

## Data Availability

The original contributions presented in this study are included in the article. Further inquiries can be directed to the corresponding author.

## Supplementary Materials

The following supporting information can be downloaded at https://www.mdpi.com/article/10.3390/nu18172790/s1, Figure S1: Rarefaction curves of gut microbial communities. The Sobs index is plotted against sequencing reads. Curves approaching saturation indicate sufficient sequencing depth across samples; Table S1: Species diversity and richness of mouse colon contents.
