# Supplementary material for: Probiotic–Herb–Peptide Combined Formula Alleviates Alcoholic Liver Damage Associated with Modulation of the Gut–Liver Axis and Ferroptosis
Source: Nutrients. 2026 Aug 26;18(17):2790. doi: 10.3390/nu18172790 (PMC13567178; doi:10.3390/nu18172790)
Supplement: Supplementary file 1 [file nutrients-18-02790-s001.zip › nutrients-4473177-supplementary.pdf]

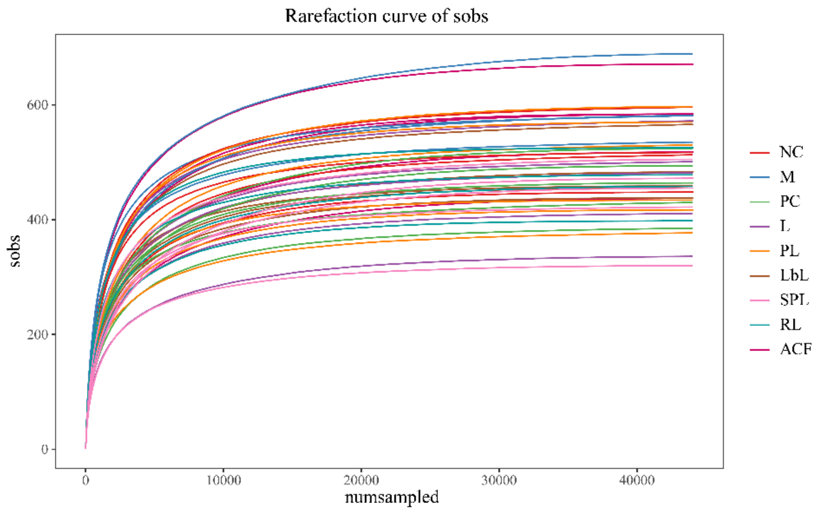

Supplemental Figure S1. Rarefaction curves of gut microbial communities. The Sobs index is plotted against sequencing reads. Curves approaching saturation indicate sufficient sequencing depth across samples.

Supplemental Table S1. Species diversity and richness of mouse colon contents.

Graph	Chao	Ace	Shannon	Invsimpson
NC	521.45±62.27	521.29±62.24	4.59±0.46	38.98±24.10
M	431.28±90.54	430.62±89.86	4.47±0.57	44.05±37.62
PC	461.23±52.70	461.77±52.82	4.46±0.24	33.90±14.54
L	466.50±94.78	464.77±92.50	4.51±0.35	37.43±14.95
PL	488.70±89.85	489.18±89.78	4.67±0.22	44.93±12.03
LbL	484.08±55.71	483.02±52.67	4.53±0.22	32.36±9.95
RL	464.78±52.58	465.00±52.42	4.77±0.30	55.68±19.70
SPL	520.84±62.07	521.41±61.82	4.52±0.51	38.11±35.66
ARF	609.52±70.07	610.66±70.61	5.05±0.20	62.87±10.81
